# A novel gene expression-based prognostic scoring system to predict survival in gastric cancer

**DOI:** 10.18632/oncotarget.10533

**Published:** 2016-07-11

**Authors:** Pin Wang, Yunshan Wang, Bo Hang, Xiaoping Zou, Jian-Hua Mao

**Affiliations:** ^1^ Department of Gastroenterology, Drum Tower Clinical Medical School Of Nanjing Medical University, Nanjing, Jiangsu 210008, China; ^2^ Biological Systems and Engineering Division, Lawrence Berkeley National Laboratory, Berkeley, CA 94720, USA; ^3^ International Biotechnology R&D Center, Shandong University School of Ocean, Weihai, Shandong 264209, China

**Keywords:** gene biomarkers, prognostic score, gastric cancer

## Abstract

Analysis of gene expression patterns in gastric cancer (GC) can help to identify a comprehensive panel of gene biomarkers for predicting clinical outcomes and to discover potential new therapeutic targets. Here, a multi-step bioinformatics analytic approach was developed to establish a novel prognostic scoring system for GC. We first identified 276 genes that were robustly differentially expressed between normal and GC tissues, of which, 249 were found to be significantly associated with overall survival (OS) by univariate Cox regression analysis. The biological functions of 249 genes are related to cell cycle, RNA/ncRNA process, acetylation and extracellular matrix organization. A network was generated for view of the gene expression architecture of 249 genes in 265 GCs. Finally, we applied a canonical discriminant analysis approach to identify a 53-gene signature and a prognostic scoring system was established based on a canonical discriminant function of 53 genes. The prognostic scores strongly predicted patients with GC to have either a poor or good OS. Our study raises the prospect that the practicality of GC patient prognosis can be assessed by this prognostic scoring system.

## INTRODUCTION

Gastric cancer (GC) is a malignant tumor initiated from the epithelial cells of gastric mucosa. GC has been one of the most common malignant tumors in the world and ranks fifth in the incidence rate, following lung cancer, breast cancer, colorectal cancer and prostate cancer [1, 2]. Despite of the slightly reduced overall incidence and mortality of GC over the past decade, to date, the incidence and mortality of GC still remain very high. Moreover, the number of people suffering from GC follows an upward trend, and there are about one million of new cases each year [1, 2]. With the advances in science and biotechnology, the level of early diagnosis for GC has been improved to certain extent, which, in turn, significantly improves its five-year survival rate. Even so, the five-year survival rate of advanced GC is only about 29.3%, which is due to that GC is prone to relapse and metastasis [1–4].

GC is a polygenic disease, where the interactions of various cancer genes with the microenvironment *in vivo* lead the early lesions of gastric mucosa to the dysplasia, and ultimately to the development of GC. The characteristically differential expression of related genes can be observed throughout the whole process. In clinical practice, there has been a lack of corresponding molecular markers for the distinguishment of GC staging and degree of differentiation. Through analyzing the GC and adjacent normal tissues using the microarray technology, Cui *et al* [5] first established a gene expression profile related to GC staging and differentiation. A total of 19 genes were involved in the expression profile, which was reported to be able to distinguish the highly differentiated and poorly differentiated GC with a relatively high accuracy rate. In addition, they also discovered a series of gene expression profiles capable of identifying GC staging.

In recent years, researchers also proposed that the molecular biological characteristics in GC composition play an important role in the prognosis. Currently, the overexpression of *HER2* gene is closely associated with the prognosis and lymph node metastasis of GC [6]. p53 is a broadly studied tumor suppressor associated with malignancy, and the accumulation of this protein in GC has also been confirmed and appears to be negatively correlated with the prognosis. By determining p53 in plasma and stomach tissue, Mattioni *et al* [7] found that the survival rate of patients with positive anti-p53 expression in plasma was significantly higher than those with the negative result. The transcription factor hypoxia-inducible factor 1 alpha (HIF-1α) is highly expressed in GC cells and exhibits an even higher expression in patients with GC at the early stage as identified by TNM classification. Therefore, HIF-1α may be related to the early development of GC and understanding its function may be helpful in exploring GC origin [8].

In the current cancer research, there are still certain difficulties in analyzing the biological significance of most genes. The gene expression profiling technology is of great significance for the investigation of different subtypes and their prognosis, and the construction of genes into a network with the help of gene expression profiling technology proves to be critical for the understanding of cancer initiation and development. Based on the analysis on the transcriptional profiles of GC at different stages, Takeno *et al.* [9] constructed a GC regulatory network with CDKNIA as the node and screened out seven genes related to GC occurrence (*i.e*., MMP7, SPARC, SOD2, INHBA, IGFBP7, NEK6 and LUM); through dividing these seven genes into two groups according to their correlation with expression levels and stages, the results showed that these seven genes were activated as the disease progressed, indicating that these genes may be associated with cancer development.

In this study, we tested the hypothesis that molecular features of GC are a key driver of tumor behavior, which can be used to establish prognostic scoring system that can improve prediction of clinical outcome. We first established a multi-step analytic approach to identify a comprehensive panel of gene biomarkers using publically available and well-characterized dataset and TCGA (The Cancer Genome Atlas), and then we employed different multivariate clustering techniques to identify the key genes for prognostic classification. Based on these analyses, we created a 53-gene expression prognostic scoring system and successfully applied it to predict overall survival (OS) in the TCGA GC as well as the GSE15459 data.

## RESULTS

### Identification of robust differentially expressed genes in gastric cancers

**Figure 1:**
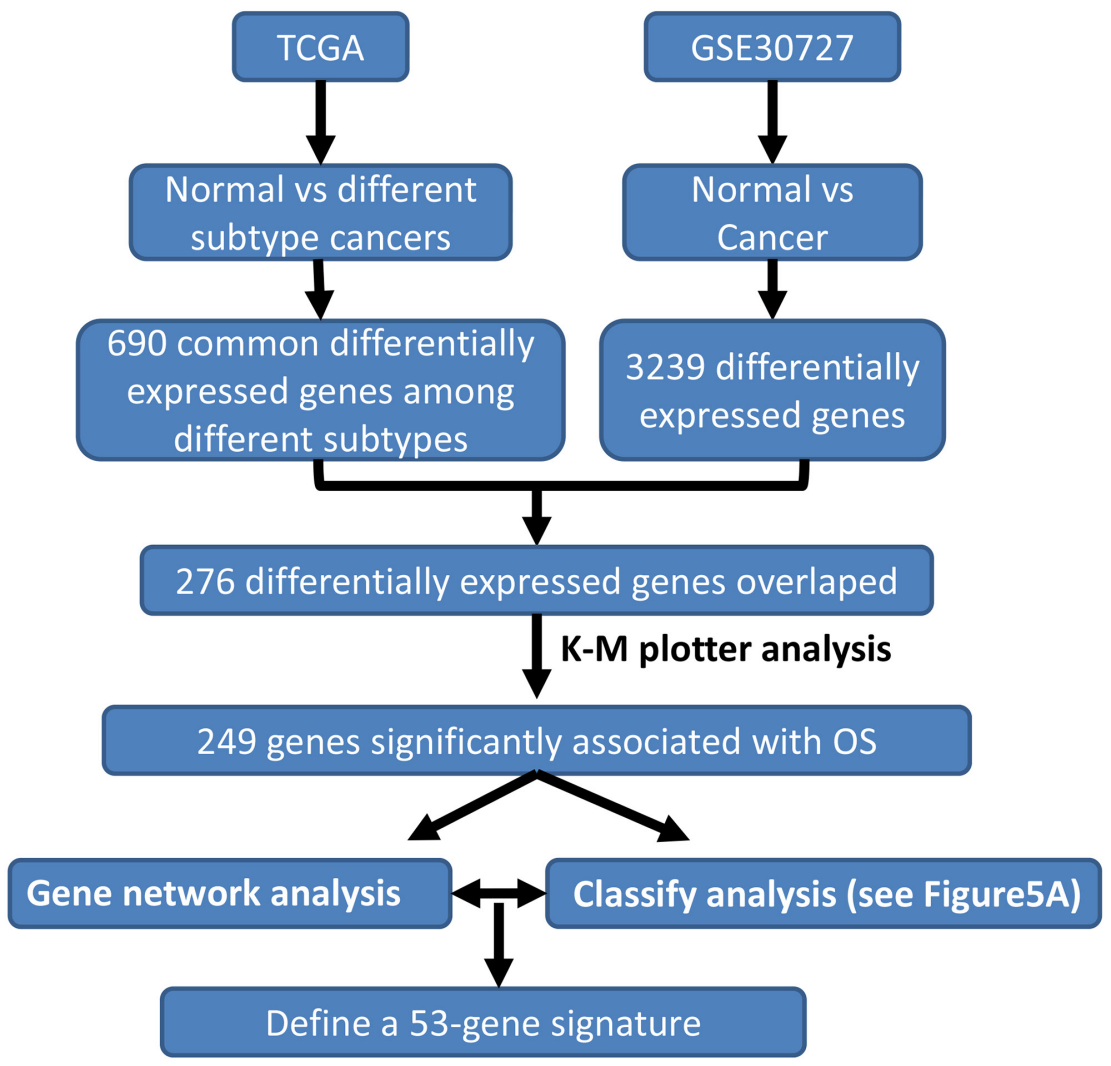
Schematic diagram for a multi-step strategy to identify gene signature for prognosis in gastric cancer The results for each step have been summarized.

We developed a multi-step strategy to identify a critical gene signature that is able to distinguish good and bad prognosis for GC patients using publically available datasets (Figure 1). Firstly, we sought to identify significantly differentially expressed genes through comparing gene expression between normal and GC tissues using two datasets: TCGA that was generated by RNA sequencing [10] and GSE30727 that was generated by Affymetrix microarray (http://www.ncbi.nlm.nih.gov/geo/query/acc.cgi?acc=GSE30727). A total of 688 and 3239 genes reached our criteria (2 fold changes and adjusted p-value <0.05) in TCGA and GSE30727, respectively (Supplementary Table S1). Importantly, 276 genes were found to be overlapping between TCGA and GSE30727 datasets. Of which, 57 genes were found to be downregulated while 219 genes were found to be upregulated in GCs (Supplementary Table S2).

### Evaluation of prognostic impact of differentially expressed genes in gastric cancers

**Figure 2:**
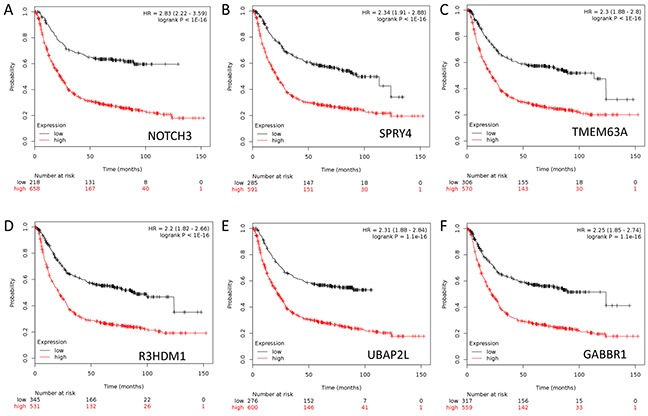
Kaplan-Meier survival curves for gastric cancer patients according to tumor expression of top rank 6 genes are presented **A.** NOTCH3. **B.** SPRY4. **C.** TMEM63A. **D.** R3HDM1. **E.** UBAP2L. **F.** GABBR1. The p values were obtained from a log-rank test between two groups.

To further assess the importance of the above 276 genes in GC development, we next evaluated their prognostic value for GC patients in a large public clinical microarray database using the Kaplan-Meier plotter (http://kmplot.com/analysis/index.php?p=service&cancer=gastric) [11]. Each of these genes was divided into two groups based on its expression value. Subsequently, the effects of high or low expression level of these genes on the overall survival (OS) were evaluated using Cox regression analysis, the Kaplan-Meier survival curve and log-rank test. 249 out of 276 genes were found to be significantly associated with OS (Supplementary Table S3). This result suggested that these molecular markers may provide a prediction for the prognosis of GC patients. Finally we ranked the genes according to their log-rank test p-values derived from univariate analysis (Supplementary Table S3), which served as the criteria for the choice of genes into canonical discriminant function (see below). Figure 2 showed the Kaplan-Meier curves for top six genes in GCs.

### Creation of gene co-expression networks for 249 genes in gastric cancer

**Figure 3:**
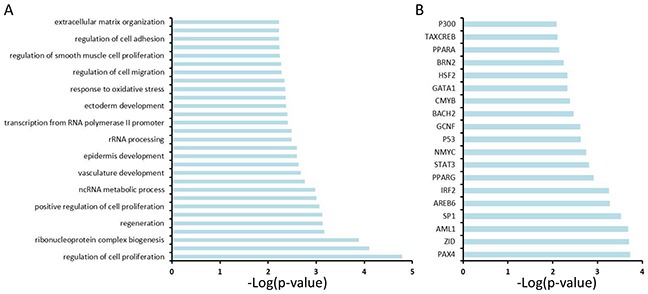
Functional annotation analysis of 249 genes using the Database for Annotation, Visualization and Integrated Discovery (DAVID) **A.** DAVID analysis reveals the potential signaling pathways that are enriched among 249 genes. **B.** DAVID analysis reveals upstream transcriptional factors that regulate the expression of 249 genes.

In order to better reveal the molecular mechanism underlying GC development, we computationally mapped the 249 genes to biological functions, pathways and upstream transcriptional regulators using the Database for Annotation, Visualization and Integrated Discovery (DAVID), and observed that these genes are significantly enriched for regulating cell proliferation, adhesion and migration, RNA/non-coding (nc) RNA process, extracellular matrix organization, vasculature development, response to oxidative stress, *etc.* (Figure 3A), all of which are hallmarks of cancer. Analysis of the upstream regulators of these 249 genes suggests that the NMYC, STAT3, GATA1 and p53 pathways play a role in GC (Figure 3B).

**Figure 4:**
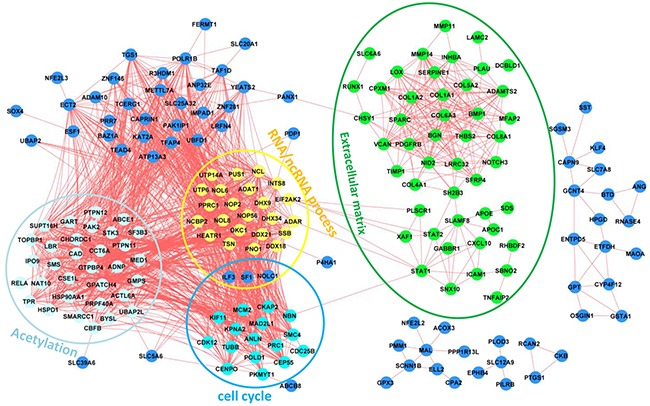
Gene correlation networks of the 249 genes Functional annotations have been indicated for different subnetworks.

Sets of genes that exhibit correlated expression patterns often share a common function or are part of the same physical structure. We next used a network analysis approach to identify functionally related groups of genes using TCGA data, which contain 265 patients with GC (Supplementary Table S4) [10]. We started by representing TCGA GC expression as a network where significantly correlated genes are drawn as nodes connected by an edge (FDR<0.05 and |r| ≥0.7; details see Materials and Methods). We then identified fully connected gene sets (cliques) that were enriched for functions (Figure 4). These sub-networks were enriched for genes representing hallmarks of cancer as discovered in the Figure 3A. Interestingly, the subnetworks for cell cycle, RNA/ncRNA process and acetylation are highly connected with each other, in contrast to the subnetwork for extracellular matrix (Figure 4).

### Development of a 53-gene prognostic scoring system

**Figure 5:**
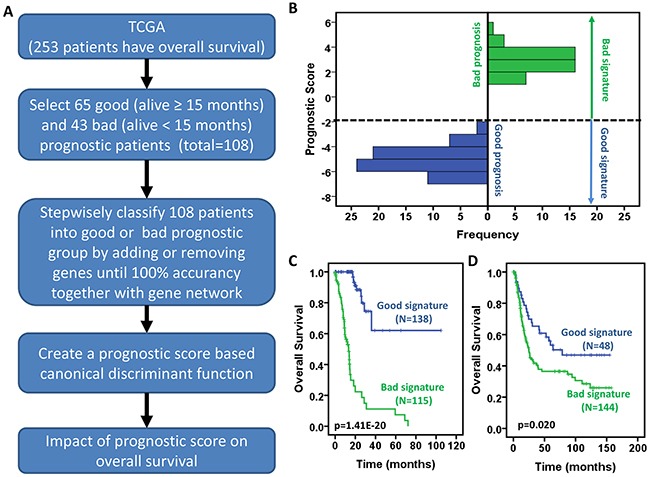
Development of a prognostic scoring system for gastric cancer patients **A.** Schematic diagram for a multi-step strategy to develop a prognostic scoring system for gastric cancer patients using the TCGA data. **B.** Distribution of prognostic score between patients with good and bad prognosis. **C-D.** Prognostic scores are significantly associated with overall survival of gastric cancer patients in TCGA GC data (C) and GSE15459 (D). Kaplan-Meier survival curves for gastric cancer patients according to prognostic scores. The p values were obtained from a log-rank test between two groups.

We designed a strategy to develop a prognostic scoring system (Figure 5A). Firstly 65 good (status is alive and time of follow-up ≥ 15 months) and 43 bad (status= deceased and the time of survival < 15 months) prognostic patients (total 108 patients) were selected as a training set from 253 of 269 GC patients who have the information of OS and OS status in a TCGA-based study (Supplementary Table S4) [10]. Then we applied a stepwise canonical discriminant analysis to identify a gene signature that is able to classify 108 patients into good or bad prognosis with 100% accuracy. To determine the optimal number of genes in a given sub-network used for building the signature, combinations of genes were tested by adding one gene at a time according to their ranks given above. The number of significant genes that gave the highest discriminative ability between good and bad prognostic groups was considered optimal. The same procedure was executed for those genes that were not in the gene co-expression network. Finally we identified a 53-gene signature to yield 100% accuracy to separate 108 patients into good or bad prognosis. A prognostic score for a patient was used to calculate a patient's risk of death and was defined as the linear combination of logarithmically transformed gene expression levels weighted by canonical discriminant function coefficients (Supplementary Table S5). The distributions of prognostic score in good and bad prognostic patients was clearly discriminative (Figure 5B), indicating that this prognostic scoring system has its discriminative ability to distinguish good prognostic patients from bad prognostic patients.

### Prediction of overall survival in gastric cancer patients

The above-developed prognostic scoring system was applied to all 253 GC patients who have the information of OS and OS status in a TCGA-based study [10]. Prognostic score was used to predict survival probability for each individual patient. We divided patients into two groups named good and bad signature based on prognostic score. If the prognostic score is ≤ −2, we defined that the patient had good signature; if the prognostic score is >-2, we defined the patient as bad signature. As shown in Figure 5C, the patients with good signature had significantly longer overall survival than those with bad signature. More than 50% of patients with good signature still survived after 100 months while all patients with bad signature died before 80 months. Next, we validated this prognostic scoring system using the GSE15459 public dataset (Supplementary Table S6) [12]. Although the GC tissues were profiled by Affymetrix microarray, which caused the difference in baseline and expression level scale, the prognostic score still predicted prognosis, the patients with lower score (good signature, the 1st quartile) significantly survived longer than the others (bad signature, the 2nd – 4th quartiles) (Figure 5D). These results raise the prospect that the practicality of gastric cancer patient prognosis can be assessed by this prognostic scoring system.

## DISCUSSION

GC is one of the leading causes of death among women worldwide [1, 2]. A growing body of evidence has demonstrated that GC is a complex and heterogeneous disease with substantial variation in their molecular and clinical characteristics. Microarray and next generation sequencing technologies have been invaluable tools to deconvolute the heterogeneity and complexity of somatic GC genetics, providing tremendous information to define new biomarkers for diagnosis, prognosis and prediction of therapeutic response, and to identify new potential therapeutic targets. Several molecular characterization studies have been conducted in GC [10, 13–15], including few that have attempted to identify the gene signature for prognosis in GC [12, 16–18]. However, there have been no reports on a prognostic scoring system that can be practically used in preclinical and clinical research. In this study, we have developed a multi-step strategy to define a 53-gene signature for prediction of overall survival for GC patients using the multi-omics data, and for the first time established a prognostic scoring system based on such a 53-gene signature. We also showed that the prognostic scores are able to distinguish patients with good prognosis from those with bad prognosis.

It is not surprising to find that among 53 genes, some of them have already been implicated in GC in previously published studies, including providing useful prognostic information about the survival. These genes include *TNFAIP2* [19], *FGFR4* [20–22], *CXCL10* [23], *CEP55* [24], *CXCL1* [25, 26], *LIMK1* [27], *LAMC2* [28], *APOE* [29], *INHBA* [30], *OSMR* [31, 32], *APOC1* [33], *KLF4* [34], *MMP14* [35], *ADH1C* [36], *COL6A3* [37, 38], *CCT2* [39], *NOL8* [40], *EPHB4* [41] and *MCM2* [42, 43]. Ye *et al* reported that high expression of FGFR4 protein is associated with a poor prognosis in patients with advanced GC and expedites the progress of advanced GC [20]. Moreover, Shen *et al* found that the FGFR4 Gly388Arg polymorphism is a useful prognostic marker for GC patients when the tumor is relatively small, well differentiated, or at an early clinical stage [21]. CEP55 is one of the centrosome family proteins and functions in cell cycle regulation, which is one of the cancer hallmarks. Knockdown of CEP55 led to reduced proliferation and colony formation in SGC7901 and BGC823 cell lines through affecting the PI3K/AKT signal pathway and the expression of cyclin-related proteins, suggesting that CEP55 might be a potential therapeutic target for GC [24]. Consistent with our finding, some studies showed that MCM2 expression levels predict diagnosis and prognosis in GC [42, 43]. miRNAs are a vital and evolutionarily ancient component of gene regulation. SNPs in miRNA binding sites could affect its binding affinity to target genes. A recent study showed that the TNFAIP2 miRNA binding site rs8126 T>C SNP may be a marker for susceptibility to GC [19].

Although the function and role of some other genes in the 53 genes selected have not been reported for their association with GC, their importance as cancer genes have been demonstrated in many studies with other types of human cancers. For example, Chemokine (C-X-C motif) ligand (CXCL1) plays a critical role in tumor metastasis and is demonstrated to be significantly associated with Snail expression. It has been reported that expression of *CXCL1* is associated with hepatocellular carcinoma survival [25]. Our study showed that *CXCL1* is implicated in GC overall survival. ABCE1 (ATP-binding cassette E1) plays a crucial role in the metastasis and progress of lung cancers, and therefore it has been suggested as a valuable therapeutic target for the management of these cancers [44]. We have demonstrated in this study that ABCE1 is elevated in GC and is an indicator for the prognosis of GC.

In summary, using the available multi-omics data, we have generated a prognostic scoring system that has been demonstrated successful prediction of patient overall survival in TCGA and a microarray dataset. Knowing the accurate prognosis of a patient with GC is very important for the determination of a most suitable clinical therapy for the patient. Clearly further studies are needed to establish the clinical application of this prognostic scoring system for GC.

## MATERIALS AND METHODS

### Datasets used in this study

The differentially expressed genes were assessed in the TCGA [10] and microarray dataset (GSE30727) profiled with Human Exon 1.0 ST Array (HuEx-1_0-st). The process data for GSE15459 from GEO website were downloaded for analysis. The expression levels of 276 genes for the set of samples in each TCGA study were obtained from the cBioPortal for cancer genome (Supplementary Table S4) [45, 46].

### Gene co-expression network construction

A network of 249 genes was constructed based on the Expression Correlation software tool (http://baderlab.org/Software/ExpressionCorrelation). Correlation coefficients exceeding a threshold (|r|≥0.7) and false discover rate (FDR <0.05) were displayed as ‘edges’ between two ‘nodes’ (where nodes represent genes), and this approach was used to define the 249 genes co-expression network. The gene co-expression network figure was generated using Cytoscape version 2.8.0 (www.cytoscape.org). The transcriptional network of the 249 genes was assessed using the data from TCGA [10].

### Statistics analysis

GEO2R was used to identify the differentially expressed genes between normal and tumor tissues in GSE30272 (adjusted p-value ≤0.05 and fold changes ≥2). The list of differentially expressed genes in TCGA was obtained from the published study [10].

The association of 276 genes with overall survival of GC patients was assessed using the Kaplan-Meier plotter (http://kmplot.com/analysis/index.php?p=service&cancer=gastric). The log-rank test p-values derived from Kaplan-Meier analysis were used to rank the gene.

108 patients in TCGA study [10] were selected as a training set using two-step stratified sampling methods. In the first step, patients were divided into two groups based on their survival status (alive *VS* deceased), and in the second step, patients in each group were further divided based on their survival time (<15 months or ≥15 months).

After identifying the relevant genes and their influence upon the prognostic model in 108 training samples, we established a prognostic scoring system as following:

$$\text{Prognostic score}=\sum_{i=1}^{53}\left(\text{Canonical Discriminant Function Coefficient})*(\text{gene i expression level}\right)$$

And then the individuals of the populations were assigned a prognostic score to stratify them into two subgroups of prognostic relevance. In this preliminary work, the groups were assigned according to prognostic score as following:

If the prognostic score ≤ −2, we defined the patient with good signature; if the prognostic score > −2, we defined the patient with bad signature. This helped to ultimately stratify the cohorts of patients in a Kaplan–Meier curve. All statistical analyses were performed using the Statistical Package for the Social Sciences version 11.5 (SPSS, Inc., Chicago, IL).

## SUPPLEMENTARY TABLES












